# Fecal Zonulin-Related Proteins in Inflammatory Bowel Disease: Associations with Clinical Disease Activity and Inflammatory Markers

**DOI:** 10.3390/medicina62061160

**Published:** 2026-06-15

**Authors:** Sergiu Ioan Frandeș, Oana Frandeș, Melania Macarie, Claudiu Marinel Ionele, Simona Maria Bățagă

**Affiliations:** 1 Doctoral School of Medicine and Pharmacy, George Emil Palade University of Medicine, Pharmacy, Science, and Technology of Târgu Mureș, 540139 Târgu Mureș, Romania; 2 Department of Internal Medicine I, George Emil Palade University of Medicine, Pharmacy, Science, and Technology of Târgu Mureș, 540139 Târgu Mureș, Romania; 3 Department of Gastroenterology, University of Medicine and Pharmacy of Craiova, 200349 Craiova, Romania

**Keywords:** fecal zonulin, inflammatory bowel disease, Crohn’s disease, ulcerative colitis, intestinal permeability, biomarkers

## Abstract

*Background and Objectives*: Intestinal barrier dysfunction is increasingly recognized as a contributor to inflammatory bowel disease (IBD) pathophysiology. Zonulin, a regulator of epithelial tight-junction permeability, has emerged as a potential non-invasive biomarker; however, its clinical relevance remains uncertain. This study evaluated whether fecal zonulin levels reflect clinical disease activity in inflammatory bowel disease and explored their association with ileal involvement in Crohn’s disease (CD). *Materials and Methods*: Forty-six consecutive IBD patients (26 CD, 20 UC) were prospectively included. Fecal zonulin was measured using a commercially available ELISA. In this study, the term “fecal zonulin” refers to ELISA-detected zonulin-related proteins. Clinical disease activity was assessed using CDAI for CD and the Mayo score for UC. Standard blood and fecal inflammatory markers were obtained, and subgroup analyses were performed according to disease type and location. *Results*: Fecal zonulin levels were significantly higher in active IBD compared with remission (106.37 vs. 53.80 ng/mL, *p* = 0.002). Patients with CD had higher zonulin concentrations than those with UC (91.4 vs. 51.0 ng/mL, *p* = 0.001). Zonulin showed a moderate positive correlation with fecal calprotectin (r = 0.338; *p* = 0.021). In multivariable analysis, clinical disease activity remained independently associated with zonulin levels, whereas ileal involvement was no longer statistically significant. *Conclusions*: Fecal zonulin is associated with disease activity in IBD, suggesting that fecal zonulin-related proteins may represent a potential adjunctive marker of epithelial barrier dysfunction and clinical disease activity in IBD. However, these findings should be considered exploratory and require validation in larger, longitudinal multicenter studies using standardized assays and endoscopic correlation.

## 1. Introduction

Inflammatory bowel diseases (IBD), mainly represented by Crohn’s disease and ulcerative colitis, are characterised by a chronic inflammatory process in the gastrointestinal tract. In ulcerative colitis, the inflammation is limited to the mucosa of the colon and/or rectum. In contrast, in Crohn’s disease, any segment of the digestive tract can be affected with discontinuous and transmural involvement [1,2]. The predominant clinical manifestations differ between the two entities, with diarrhea and rectal bleeding being more common in ulcerative colitis, and abdominal pain and weight loss being predominant in Crohn’s disease.

Inflammatory bowel diseases are chronic conditions, with a prevalence of 200–400 cases per 100,000 inhabitants. Their prevalence is continuously increasing globally, with the number of cases increasing from approximately 3.7 million in 1990 to 6.8 million cases in 2017 [1,3].

The pathogenesis of inflammatory bowel diseases remains unknown; the latest hypothesis suggests that IBD results from an abnormal immune response to the intestinal commensal flora in genetically susceptible patients [1,4]. Alterations in the functionality of the intestinal barrier increase epithelial permeability, allowing luminal antigens to pass and interact with the mucosa-associated immune system. This disruption of immune tolerance mechanisms and the maintenance of homeostasis may favor the initiation and perpetuation of certain aberrant immune responses, contributing to the development of a chronic inflammatory state and the pathogenesis of diseases with an immunological component [1,5].

Zonulin is a human protein with a molecular mass of 47 kDa that plays a role in increasing the permeability of the epithelial layer of the small intestine. It is considered one of the best-characterized endogenous modulators of intestinal permeability. The normal functionality of these junctions is essential for the physiological processes in the intestine [6,7]. Recent studies have demonstrated that zonulin does not represent a single molecular entity but rather a group of structurally and functionally related proteins derived from the precursor protein haptoglobin-2 (pre-HP2). Consequently, the use of the term “zonulin-related proteins” (ZRP) is recommended to more adequately reflect this molecular diversity [1,8].

Zonulin (identified as pre-haptoglobin-2) increases intestinal permeability through activation of protease-activated receptor-2 (PAR2) and transactivation of the epidermal growth factor receptor (EGFR). PAR2 stimulation triggers phosphatidylinositol turnover and protein kinase C (PKC) activation. Then, PKC induces actin cytoskeleton rearrangement and phosphorylation of zonula occludens-1 (ZO-1); this reaction results in the displacement of ZO-1 from the tight junction complex and increased paracellular permeability. Through this mechanism, luminal antigens and microbial components can pass across the epithelial barrier, potentially promoting local and systemic inflammatory responses [9].

As a relatively newly discovered protein, there is little data on the involvement of the zonulin in the pathogenesis of intestinal inflammation and, therefore, of the inflammatory bowel diseases. Moreover, few aspects are published regarding the correlation between the fecal and the serum zonulin, which would be more useful for the diagnosis and the follow-up of the inflammatory bowel diseases [10].

This supports the concept that zonulin reflects intestinal barrier dysfunction rather than inflammation alone. In a recent case report, fecal zonulin levels were elevated in Crohn’s disease but not in intestinal tuberculosis, despite inflammatory involvement in both conditions [11].

The new European Crohn’s and Colitis Organization (ECCO) Guidelines for the management of Crohn’s disease emphasize the importance of the noninvasive biomarkers in the diagnosis and monitoring of the IBD activity [10,12]. Although fecal calprotectin currently remains the only validated and recommended noninvasive biomarker for the assessment of intestinal inflammation, it mainly reflects neutrophil activity and does not provide direct information on the intestinal barrier function [13,14]. In contrast, zonulin and related proteins are potential markers of intestinal permeability, but the available data on their clinical value in IBD remain limited and sometimes contradictory [15,16,17].

Although biomarkers such as C-reactive protein and fecal calprotectin are widely used in inflammatory bowel disease (IBD), they primarily reflect inflammatory burden and may have limited specificity in certain clinical settings. In addition, currently available biomarkers do not directly assess intestinal barrier dysfunction, which is increasingly recognized as an important component of IBD pathogenesis. Zonulin, a regulator of epithelial tight junction permeability, has therefore emerged as a potential non-invasive marker of altered intestinal barrier integrity. However, data regarding its clinical utility in IBD remain limited and sometimes inconsistent, highlighting the need for further investigation [18].

The primary aim of this study was to evaluate the association between fecal zonulin levels and clinical disease activity in patients with inflammatory bowel disease. Secondary exploratory analyses assessed potential differences according to disease type, disease location, inflammatory markers, and treatment categories.

## 2. Materials and Methods

The design of this study is an observational, cross-sectional, monocentric one, carried out within the Gastroenterology Clinic of the County Clinical Emergency Hospital of Târgu Mureș between June 2024 and March 2026. The study protocol was approved by the Ethics Committee of the County Clinical Emergency Hospital of Târgu Mureș (No. 10786/28 May 2024). All participants signed informed consent forms for their inclusion in the study and for the processing of their data for scientific purposes. All the performed procedures respected the ethical principles established in the Declaration of Helsinki.

This study included 46 patients with a confirmed diagnosis of inflammatory bowel disease (Crohn’s disease and ulcerative colitis) who were registered and followed up in the Gastroenterology Department of the same institution.

The inclusion criteria were: age ≥18 years, a definite diagnosis of inflammatory bowel disease, established based on clinical, endoscopic, and histopathological evaluations, as well as signing informed consent to participate in the study.

**Figure 1:**
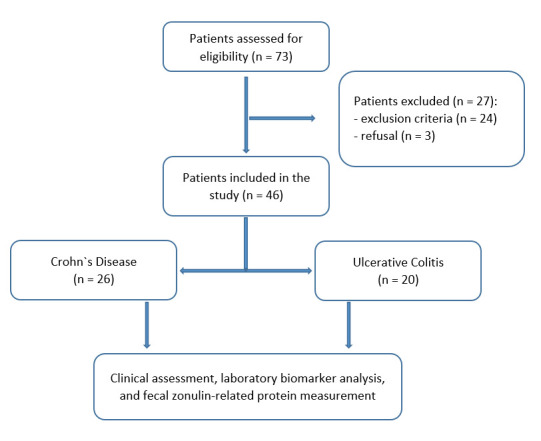
Flowchart illustrating patient selection and study inclusion.

The exclusion criteria included the presence of other chronic gastrointestinal diseases (e.g., celiac disease, gastrointestinal neoplasms, small bowel resection) that could affect intestinal permeability and zonulin levels, active infections, recent antibiotic treatment, severe comorbidities that could interfere with the study, and refusal or inability to provide informed consent. A flowchart illustrating patient selection and study inclusion is presented in Figure 1.

Patients were classified according to the Montreal classification. For Crohn’s disease, disease location was categorized as L1 (ileal), L2 (colonic), L3 (ileocolonic), or L4 (isolated upper disease). For ulcerative colitis, disease extent was categorized as E1 (proctitis), E2 (left-sided colitis), or E3 (extensive colitis).

Clinical disease activity was assessed using validated clinical indices. For Crohn’s disease, activity was defined using the Crohn’s Disease Activity Index (CDAI), with CDAI > 150 indicating active disease and CDAI ≤ 150 indicating clinical remission. For ulcerative colitis, the total Mayo score was used, with a score > 3 considered active disease and ≤3 considered remission. These definitions were consistently applied throughout the analyses.

Information regarding ongoing IBD therapy was recorded at the time of inclusion, including biologic agents, immunomodulators, corticosteroids, and aminosalicylates. For statistical analysis, treatment categories were grouped into three broader classes (5-ASA-based therapy, corticosteroid and/or azathioprine therapy, and biologic therapy) due to the small number of patients in individual subgroups.

Endoscopic evaluation was not systematically available for all patients at the time of inclusion and was performed only when clinically indicated. Therefore, clinical disease activity assessment was primarily based on clinical indices and biochemical markers.

Fecal samples were collected from all the patients included in the study and sent to the laboratory on the same day. Fecal samples were collected on the same day as clinical assessment, eliminating temporal variability between biomarker measurement and clinical disease activity.

Fecal zonulin concentrations were measured in an external accredited laboratory (Bioclinica, Târgu Mureș, Romania) using a commercial competitive enzyme immunoassay (IDK^®^ Fecal Zonulin ELISA, Immundiagnostik AG, Bensheim, Germany). Laboratory personnel had no access to patients’ clinical data, disease activity status, or study outcomes at the time of analysis. All samples were processed according to the manufacturer’s instructions. According to the technical datasheet, the assay detects zonulin-related proteins (ZRPs) rather than pre-haptoglobin-2 exclusively. The reported intra-assay coefficient of variation ranges between 4.8 and 7.5%, while the inter-assay coefficient of variation ranges between 7.1 and 12.0%. The analytical sensitivity (lower limit of detection) is approximately 1.8 ng/mL. Therefore, throughout this manuscript, the term “fecal zonulin” refers to ELISA-detected zonulin-related proteins rather than pre-haptoglobin-2 exclusively.

The reference range used in this study (<107 ng/mL) was provided by the laboratory and is based on the manufacturer’s validation studies conducted in apparently healthy individuals.

Concomitantly with stool sample collection, 10 mL of venous blood was collected from patients included in the study. Laboratory parameters, including hemoglobin, hematocrit, neutrophil count, lymphocyte count, monocyte count, platelet count, iron status, total protein, serum albumin, C-reactive protein, erythrocyte sedimentation rate, and calprotectin, were recorded in the study database. All measurements were performed in the laboratory of the Târgu Mureș County Emergency Hospital by individuals other than the study authors. The reference intervals for the above-mentioned parameters provided by the laboratory are presented in Table 1.

Statistical analysis was performed using IBM SPSS Statistics (version 26.0). Normally distributed continuous variables were expressed as mean ± standard deviation (SD), while non-normally distributed variables were expressed as median and interquartile range (IQR). Categorical variables were presented as absolute numbers and percentages. The distribution of continuous variables was assessed using the Kolmogorov–Smirnov test.

Correlations between variables were analyzed using Spearman’s rank correlation coefficient for non-normally distributed variables and Pearson’s correlation coefficient for normally distributed variables. Differences between groups were evaluated using Student’s *t*-test for normally distributed continuous variables and the Mann–Whitney U test for non-normally distributed variables. The Kruskal–Wallis test was used to compare continuous variables across multiple groups, followed by Bonferroni-adjusted pairwise comparisons.

Linear regression analysis was performed to assess the relationship between laboratory parameters and to identify independent predictors of fecal zonulin levels.

To evaluate the potential of fecal zonulin as a biomarker for discriminating disease status, receiver operating characteristic (ROC) curve analysis was performed, with calculation of the area under the curve (AUC), sensitivity, and specificity. The optimal cut-off values were determined using the Youden index.

A *p*-value < 0.05 was considered statistically significant.

## 3. Results

### 3.1. Patient Characteristics

A total of 46 patients with inflammatory bowel disease (IBD) were included in the analysis. The mean age of the cohort was 41.3 ± 15.2 years (range 20–75), and 60.9% were male. Regarding disease type, 26 patients (56.5%) had CD, and 20 (43.5%) were diagnosed with UC. According to the Montreal classification, Crohn’s disease patients were distributed as follows: L1 (n = 3), L2 (n = 8), and L3 (n = 15). Ulcerative colitis patients were classified as E1 (n = 1), E2 (n = 14) or E3 (n = 5). According to clinical disease activity assessment, 18 (39.1%) patients presented active disease, whereas 28 (60.9%) were in clinical remission. Table 2 summarizes the treatment characteristics of the study population.

For readability, the term ‘fecal zonulin’ is used throughout the Results and Discussion sections to refer to ELISA-detected zonulin-related proteins.

Median age was 39 years (IQR 29–50). Median hematocrit was 40.25% (IQR 36.77–44.92), while mean hemoglobin was 13.25 ± 2.22 g/dL. Leukocyte counts were generally within reference ranges, with median neutrophil and monocyte counts of 4.63 × 10^9^/L (IQR 3.68–5.53) and 0.57 × 10^9^/L (IQR 0.44–0.74), respectively, whereas mean lymphocyte count was 2.17 ± 0.80 × 10^9^/L. Median platelet count was 316 × 10^9^/L (IQR 257–368). Median inflammatory marker levels were elevated, with CRP of 3.82 mg/L (IQR 1.8–9.56) and ESR of 10.5 mm/h (IQR 6.0–20.0). Median serum iron, albumin, and total protein levels were 64.10 µg/dL (IQR 29.72–95.87), 4.25 g/dL (IQR 4.02–4.60), and 7.22 g/dL (IQR 6.90–7.60), respectively. Median fecal calprotectin was 205.50 μg/g (IQR 74–704.25). Fecal zonulin levels ranged between 10 and 282.3 ng/mL, with a median concentration of 56.52 ng/mL (IQR 42.3–99.31). Descriptive statistics for laboratory parameters are summarized in Table 3.

### 3.2. Comparison of Zonulin Levels According to Clinical Disease Activity

**Figure 2:**
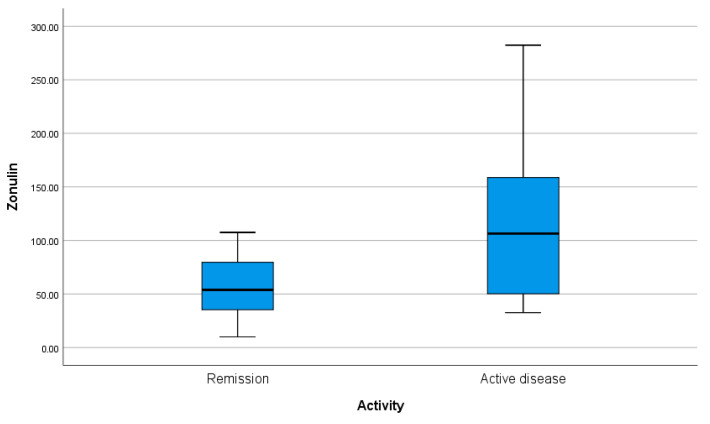
Fecal zonulin levels (ng/mL) according to clinical disease activity status in patients with inflammatory bowel disease (remission, n = 28; active disease, n = 18). Data are presented as boxplots with individual values overlaid. Zonulin concentrations were significantly higher in patients with active disease compared with remission, *p* = 0.002.

Patients were stratified according to clinical activity status (active disease vs. remission). Zonulin concentrations were significantly higher in patients with active disease compared to those in remission (106.37 (48.48–162.97) ng/mL vs. 53.80 (34.81–79.67) ng/mL, *p* = 0.002) (Figure 2). These findings suggest that zonulin levels differ according to clinical disease activity status.

### 3.3. Comparison of Zonulin Levels According to Disease Type

**Figure 3:**
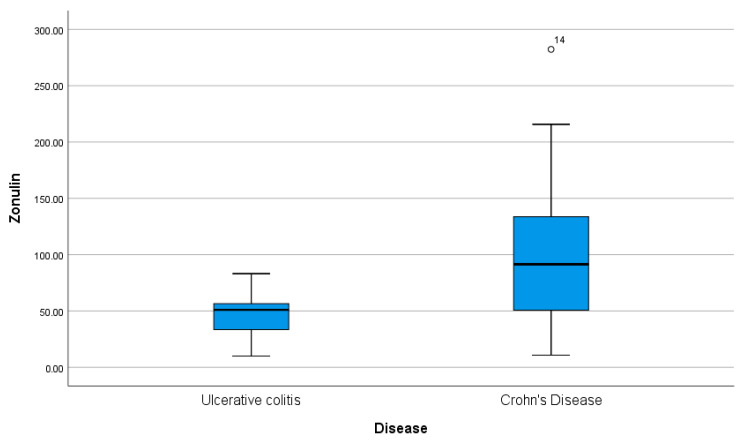
Fecal zonulin levels (ng/mL) according to disease type in patients with inflammatory bowel disease (Crohn’s disease, n = 26; ulcerative colitis, n = 20). Data are presented as boxplots with individual values overlaid. Zonulin concentrations were significantly higher in patients with Crohn’s disease compared with ulcerative colitis, *p* = 0.001.

Fecal zonulin levels differed significantly between patients with Crohn’s disease and those with ulcerative colitis. The median zonulin concentration in the Crohn’s disease group was 91.4 (48.73–136.7) ng/mL, compared to 51.0 (32.98–57.23) ng/mL in the ulcerative colitis group. The difference between groups was statistically significant (*p* = 0.001), indicating higher zonulin levels in Crohn’s disease. The Hodges–Lehmann estimated median difference was −54.56 ng/mL, suggesting that patients with Crohn’s disease tend to exhibit greater impairment of intestinal barrier function, as reflected by elevated zonulin. These findings suggest that intestinal permeability alterations may be more pronounced in Crohn’s disease than in ulcerative colitis (Figure 3).

### 3.4. ROC Curve Analysis

The discriminatory ability of zonulin was evaluated by receiver operating characteristic (ROC) analysis. The area under the curve (AUC) was 0.750 (95% CI: 0.594–0.906, *p* = 0.005), indicating moderate accuracy for distinguishing active disease from remission. The optimal cut-off point was 68 ng/mL, which provided 66.7% sensitivity and 71.4% specificity.

ROC analysis was used to evaluate the discriminatory ability of zonulin for distinguishing Crohn’s disease from ulcerative colitis. The area under the curve (AUC) was 0.777 (95% CI: 0.638–0.915, *p* = 0.001), indicating moderate accuracy. The optimal cut-off value was 68 ng/mL, yielding a sensitivity of 69.2% and specificity of 90.0%.

**Figure 4:**
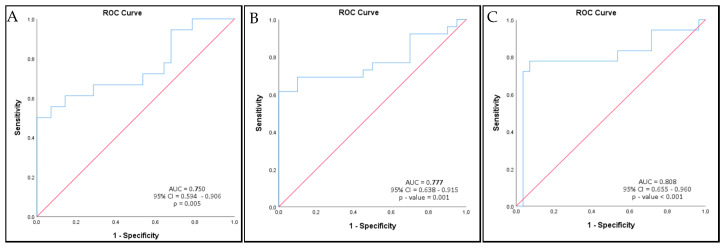
Exploratory ROC curves illustrating the discriminatory ability of fecal zonulin for distinguishing active disease (**A**), Crohn’s disease versus ulcerative colitis (**B**), and ileal involvement in Crohn’s disease (**C**).

Exploratory ROC analysis according to disease location showed moderate discriminatory ability for ileal involvement in Crohn’s disease (AUC = 0.808, 95% CI: 0.655–0.960, *p* < 0.001). The optimal cut-off value was 83.9 ng/mL, yielding a sensitivity of 77.8% and specificity of 92.9%. Figure 4 shows ROC curves showing the discriminatory ability of fecal zonulin for distinguishing clinical disease activity, Crohn’s disease, and ileal involvement in Crohn’s disease (Table 4).

### 3.5. Linear Regression Analysis

Linear regression analysis showed that ileal involvement was significantly associated with higher zonulin levels (B = 52.001, *p* = 0.002). Ileal involvement explained 20.5% of the variability in zonulin concentrations (R^2^ = 0.205).

Multiple linear regression analysis showed that both disease type and clinical disease activity were associated with zonulin levels. Crohn’s disease was associated with higher zonulin concentrations (B = 44.814, *p* = 0.006), while active disease was associated with higher zonulin levels compared with remission (B = 35.43, *p* = 0.026). The model explained 35.7% of the variability in zonulin concentrations (R^2^ = 0.357). These analyses should be interpreted as exploratory, given the limited sample size.

In multivariate linear regression analysis, clinical disease activity was independently associated with higher zonulin levels (B = 48.687, *p* = 0.004). Ileal involvement, age, and gender were not significantly associated with zonulin levels. The model explained 39.8% of the variability in zonulin concentrations (R^2^ = 0.398, *p* = 0.001). Table 5 shows linear regression models evaluating factors associated with zonulin levels.

### 3.6. Correlation Analysis

**Figure 5:**
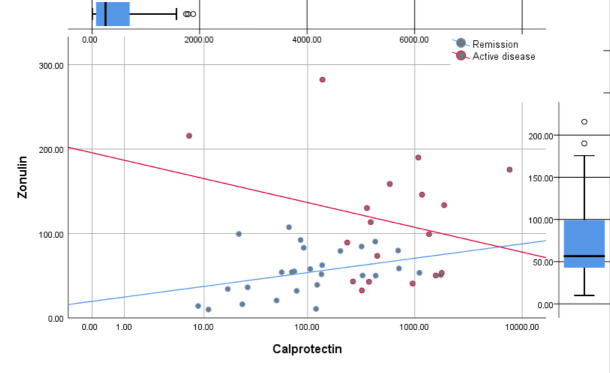
Correlation between fecal zonulin and fecal calprotectin according to clinical disease activity status (active disease, n = 18; remission, n = 28). Calprotectin values are displayed on a logarithmic scale due to their highly skewed distribution and extreme variability.

Spearman’s correlation analysis was performed to explore associations between zonulin and other laboratory parameters. Zonulin showed a moderate positive correlation with fecal calprotectin (r = 0.338, *p* = 0.021), supporting its relevance in the context of intestinal inflammatory activity (Table 6). No significant correlations were observed between zonulin and hemoglobin, hematocrit, leukocyte subsets, platelet count, CRP, ESR, serum iron, albumin, or total protein levels. The relationship between fecal zonulin and calprotectin is illustrated in the scatter plot (Figure 5), which shows an increasing trend of zonulin concentrations with higher calprotectin levels.

### 3.7. Comparison of Zonulin Levels According to Treatment Type

Fecal zonulin levels were compared across the three therapy groups: 5-ASA, Corticosteroid + Azathioprine, and Biologic therapy. The Kruskal–Wallis test was used due to the non-normal distribution of the data. No significant differences were observed between groups (*p* = 0.537), indicating that median zonulin levels did not differ significantly across treatment categories.

### 3.8. Comparison of Zonulin Levels Across Montreal Subgroups

Zonulin levels were compared across disease locations according to the Montreal classification. A global comparison using the Kruskal–Wallis test showed significant differences in zonulin concentrations between groups (103.5 (40.23–81.80) vs. 63.42 vs. 50.20 (33.99–55.89) vs. 51.70 (41.15–58.28) ng/mL; *p* = 0.015). Pairwise analyses demonstrated significantly higher zonulin levels in small bowel Crohn’s disease (L1 + L3) compared with limited ulcerative colitis (E2) (109.91 vs. 51.13 ng/mL, *p* = 0.003) and with the overall ulcerative colitis group (E1 + E2 + E3) (103.5 (40.23–81.80) vs. 51.00 (33.61–56.80) ng/mL, *p* < 0.001). In contrast, zonulin levels did not differ significantly between small bowel Crohn’s disease (L1 + L3) and colonic Crohn’s disease (L2) (103.5 (72.63–143.00) vs. 63.42 (40.23–81.80) ng/mL, *p* = 0.224). These findings are summarized in Table 7.

### 3.9. Analysis Within the Crohn’s Disease Subgroup

When Crohn’s disease patients were stratified according to ileal involvement, no significant differences in zonulin levels or other laboratory parameters were observed between the two groups.

In patients with Crohn’s disease, zonulin levels were negatively correlated with platelet count (Spearman’s r = −0.433, *p* = 0.027), indicating that higher zonulin levels were associated with lower platelet counts.

In the Crohn’s disease subgroup, linear regression analysis showed that ileal involvement was not significantly associated with zonulin levels (B = 25.8, *p* = 0.357). The model explained only 3.5% of the variability in zonulin concentrations (R^2^ = 0.035).

## 4. Discussion

The present study evaluated whether fecal zonulin levels could differentiate between active and remission phases of inflammatory bowel disease (IBD), including both Crohn’s disease (CD) and ulcerative colitis (UC). Our findings indicate that fecal zonulin concentrations were significantly elevated in patients with active IBD compared to those in remission, and that zonulin levels were higher in Crohn’s disease than in ulcerative colitis. These results support the hypothesis that increased intestinal permeability, reflected by elevated fecal zonulin, may contribute to IBD pathophysiology and clinical disease activity.

In our cohort, the median fecal zonulin concentration was significantly higher in Crohn’s disease (91.4 (48.73–136.7) ng/mL) compared to ulcerative colitis (51 (32.98–57.23) ng/mL; *p* = 0.001). This finding is biologically reasonable given that Crohn’s disease involves transmural inflammation and often affects both small and large intestines, thus causing a more pronounced impact on epithelial permeability and zonulin release.

Another theory by Arrieta et al. sustains the presence of zonulin receptors only in the small intestine [19,20]. Similar results were reported by Malickova et al., who observed higher fecal calprotectin and fecal zonulin levels in patients with active CD than in those with UC and demonstrated a positive correlation with clinical disease activity scores [6]. However, Szymanski et al. and Caviglia et al., in their studies, did not find a correlation between zonulin concentrations in patients with CD and those with UC [10,17]. Moreover, Khusainova et al. recently confirmed that fecal zonulin levels correlate with intestinal inflammation and small bowel involvement in Crohn’s disease, suggesting that zonulin may serve as a surrogate marker of mucosal damage [3]. In that study, they showed that the zonulin concentration is directly correlated with the functional activity of circulating neutrophils. Exploratory subgroup analyses based on the Montreal classification showed significant overall differences in zonulin levels across subgroups; however, these findings should be interpreted cautiously given the limited sample size of several categories.

In the fully adjusted linear regression model, clinical disease activity remained independently associated with fecal zonulin levels (B = 48.69, β = 0.424, *p* = 0.004), while ileal involvement and disease type were not statistically significant. Previous studies have also reported an association between fecal zonulin and clinical disease activity [6,10,21]. In contrast, Wegh et al., who investigated markers of intestinal permeability in ulcerative colitis, found that serum, rather than fecal, zonulin levels correlated more strongly with CRP [16]. In our study, higher fecal zonulin levels were associated with active disease, supporting its potential role as a marker of clinical disease activity. Although the discriminative power of zonulin, as reflected by the ROC curve (AUC = 0.750), was moderate, this finding is consistent with previous reports suggesting that zonulin alone is not sufficient for diagnostic purposes but may have additive value in multimarker panels.

In our study, we analyzed the possible correlation between zonulin and systemic inflammatory markers; however, no significant correlations were observed between zonulin and hemoglobin, hematocrit, leukocyte subsets, platelet count, CRP, ESR, serum iron, albumin, or total protein levels. In their studies, Said et al. and Tatucu-Babet et al. reported a positive correlation between zonulin and TNF-α and hs-CRP with significant *p*-values in their cohort [22,23].

In our study, we observed a moderate positive correlation between zonulin and fecal calprotectin. This association suggests that increased intestinal permeability parallels mucosal inflammatory activity. Although zonulin is not a direct inflammatory biomarker, its correlation with calprotectin supports its relevance as a marker reflecting clinical disease activity through epithelial barrier dysfunction. These findings are consistent with previous reports showing an association between fecal zonulin levels and markers of intestinal inflammation [10,21,24].

Previous studies suggest that biologic therapies may improve intestinal barrier function in parallel with mucosal healing in IBD, although available data regarding their effects on zonulin-related proteins remain limited [25,26,27].

If validated in larger prospective studies, fecal zonulin-related proteins could potentially complement currently available biomarkers by providing additional information regarding intestinal barrier dysfunction. In the future, such markers may have utility as part of multimarker approaches integrating inflammatory, permeability, and clinical parameters. Current evidence remains insufficient to support routine clinical implementation.

Due to the limited sample size and the heterogeneous distribution of biologic therapies, the study was not powered to evaluate the independent effect of biologic agents on fecal zonulin concentrations. This remains an important limitation and should be addressed in future studies with stratified treatment groups.

This study has several limitations that should be acknowledged. First, the sample size was relatively small (n = 46), reflecting the characteristics of our small-volume tertiary center and the limited pool of eligible IBD patients. Although subgroup analyses were exploratory due to the small sample sizes, the observed differences between Crohn’s disease and ulcerative colitis remain clinically meaningful. The heterogeneous distribution of biologic therapies also restricted our ability to evaluate their independent effects on fecal zonulin levels. Detailed subgroup analyses according to biologic class were not feasible due to the limited sample size.

Second, measuring zonulin reliably remains challenging. Current ELISA kits may detect a broader family of zonulin-related proteins rather than pre-haptoglobin-2 alone, potentially affecting quantitative accuracy. In addition, potential confounders—such as diet, microbiota composition, medication use, and extraintestinal inflammation—were not systematically controlled. Finally, as a single-center study, selection bias cannot be excluded. The term “fecal zonulin” used throughout this manuscript refers to ELISA-detected zonulin-related proteins rather than pre-haptoglobin-2 exclusively, which should be considered when interpreting the findings.

Additional potential confounders, including smoking status, BMI, dietary factors, NSAID exposure, and disease duration, were not systematically assessed and may have influenced zonulin levels.

Detailed information regarding sample storage conditions, freeze–thaw cycles, and duplicate measurements was not available because analyses were performed in an external accredited laboratory.

Despite these limitations, the study provides clinically relevant real-world data from a well-characterized IBD cohort and highlights the potential value of fecal zonulin as a non-invasive marker of clinical disease activity. Future multicenter, longitudinal studies using standardized assays are needed to validate and extend these findings.

In summary, our results demonstrate that fecal zonulin is significantly associated with IBD activity and that levels are higher in Crohn’s disease compared to ulcerative colitis. Although not sufficiently specific to serve as a standalone biomarker, zonulin remains a promising indicator of intestinal permeability and epithelial dysfunction, meriting further exploration in larger, longitudinal, and mechanistic studies.

## 5. Conclusions

Our findings suggest an association between fecal zonulin-related proteins and clinical disease activity in patients with inflammatory bowel disease. Fecal zonulin-related proteins may represent a potential adjunctive marker of epithelial barrier dysfunction in IBD; however, these findings should be considered exploratory given the cross-sectional design and limited sample size. Further validation in larger, longitudinal, multicenter studies using standardized assays and endoscopic correlation is required before clinical application.

## Figures and Tables

**Table 1:** Reference intervals for laboratory parameters.

Parameter	Normal Range	Parameter	Normal Range
Hemoglobin	13–17 g/dL	Proteins	6.4–8.3 g/dL
Hematocrit	36–48%	Albumin	3.97–4.94 mg/dL
Lymphocytes count	1.2–3.4 × 10^9^/L	CRP	<5 mg/L
Neutrophils count	1.4–6.5 × 10^9^/L	ESR	1–10 mm/h
Monocytes count	0.1–0.6 × 10^9^/L	Calprotectin	<50 µg/g
Platelets	150–450 × 10^9^/L	Zonulin	<107 ng/mL
Serum Iron	33–193 µg/dL		

CRP—C-reactive protein; ESR—Erythrocyte Sedimentation Rate.

**Table 2:** Clinical and treatment characteristics of the study population.

		N	%
Gender			
	Male	28	60.9%
	Female	18	39.1%
Disease			
	Crohn’s disease	26	56.5%
	Ulcerative colitis	20	43.5%
Treatment			
	5-ASA	14	30.4%
	5-ASA + Corticosteroids	4	8.7%
	5-ASA + Azathioprine	1	2.2%
	Azathioprine	2	4.3%
	Corticosteroid	4	8.7%
	Biologic therapy	20	43.47%
	Without treatment	1	2.2%

5 ASA—5-Aminosalicylic acid.

**Table 3:** Laboratory characteristics of the study population.

Parameter	N	Distribution	Range
Age	46	39 (29–50)	20–75
Hemoglobin	46	13.25 ± 2.22 g/dL	7.5–16.3 g/dL
Hematocrit	46	40.25 (36.77–44.92) %	22.9–48%
Leucocyte profile			
Lymphocytes	46	2.17 ± 0.80 × 10^9^/L	1.12–4.99 × 10^9^/L
Neutrophils	46	4.63 (3.68–5.53) × 10^9^/L	2.57–10 × 10^9^/L
Monocytes	46	0.57 (0.44–0.74) × 10^9^/L	0.22– 1.85 × 10^9^/L
Platelets	46	316(257–368) × 10^9^/L	161–898 × 10^9^/L
CRP	46	3.82 (1.8–9.56) mg/L	0.14–113 mg/L
ESR	46	10.5 (6.0–20.0) mm/h	2–95 mm/h
Serum iron	46	64.10 (29.72–95.87) µg/dL	10.4–190.0 µg/dL
Albumin	46	4.25 (4.02–4.60) g/dL	2.37–5.0 g/dL
Total proteins	46	7.22 (6.90–7.60) g/dL	4.69–8.14 g/dL
Calprotectin	46	205.50 (74–704.25) μg/g	7.0–7630 µg/g
Zonulin	46	56.52 (42.3–99.31) ng/mL	10–282.3 ng/mL

CRP—C-reactive protein; ESR—Erythrocyte Sedimentation Rate.

**Table 4:** ROC analyses evaluating the discriminatory ability of fecal zonulin.

ROC Model	AUC (95% CI)	Cut-Off (ng/mL)	Sensitivity (%)	Specificity (%)	*p*-Value
Active disease vs. remission	0.750 (0.594–0.906)	68	66.7	71.4	**0.005**
Crohn’s disease vs. ulcerative colitis	0.777 (0.638–0.915)	68	69.2	90.0	**0.001**
Ileal involvement in Crohn’s disease *	0.808 (0.655–0.960)	83.9	77.8	92.9	**<0.001**

* Exploratory analysis performed in the Crohn’s disease subgroup.

**Table 5:** Linear regression models evaluating factors associated with zonulin levels.

Variable	B	SE	Beta	*p*
**Model 1**	**Ileal involvement**			
**Ileal involvement**	**52.001**	**15.53**	**0.453**	**0.002**
R^2^ = 0.205, *p* = 0.002				
**Model 2**	**Disease type and clinical disease activity**			
**Disease type**	**44.814**	**15.589**	**0.390**	**0.006**
**Active disease**	**35.43**	**15.348**	**0.313**	**0.026**
R^2^ = 0.357, *p* < 0.001				
**Model 3**	**Fully adjusted model**			
Ileal involvement	29.257	19.869	0.255	0.149
Disease type	15.872	20.912	0.140	0.452
**Active disease**	**48.687**	**16.007**	**0.424**	**0.004**
Gender	1.359	14.177	0.012	0.924
Age	0.380	0.475	0.102	0.429
R^2^ = 0.398, *p* = 0.001				

B = unstandardized regression coefficient; β = standardized regression coefficient; SE = standard error; *p* = significance.

**Table 6:** Correlations between fecal zonulin and hematologic, inflammatory, and biochemical markers in IBD patients.

Parameter	N	Correlation	*p*-Value
Age	46	−0.059	0.695
**Calprotectin**	46	**0.338**	**0.021**
Hemoglobin	46	0.045	0.766
Hematocrit	46	0.033	0.826
Lymphocytes	46	−0.072	0.663
Neutrophils	46	−0.011	0.944
Monocytes	46	−0.086	0.570
Platelets	46	−0.013	0.929
CRP	46	0.174	0.827
ESR	46	−0.033	0.626
Serum iron	46	0.024	0.876
Albumin	46	−0.105	0.487
Total proteins	46	0.078	0.607

CRP—C-reactive protein; ESR—erythrocyte sedimentation rate.

**Table 7:** Comparison of zonulin levels across Montreal subgroups. Data are presented as median (IQR) for comparisons analyzed using the Mann–Whitney U test or Kruskal–Wallis test, and as mean ± SD for comparisons analyzed using the unpaired t-test.

Comparison	Groups	Distribution (ng/mL)	Statistical Test	*p*-Value
Small bowel CD vs. colonic CD	L1+L3 (n = 18) vs. L2 (n = 8)	103.5 (72.63–143.00) vs. 63.42 (40.23–81.80)	Mann–Whitney	0.224
**Small bowel CD vs. limited UC**	**L1+L3 (n = 18) ** **vs. E2 (n = 14)**	**109.91 vs. 51.13**	**Unpaired ** ***t*** **-test**	**0.003**
**Small bowel CD vs. all UC**	**L1 + L3 (n = 18) vs. E1 + E2 + E3 (n = 26)**	**103.5 (72.63–143.00) vs. 51.00 (33.61–56.80)**	**Mann–Whitney**	**<0.001**
**Global comparison**	**L1 + L3 (n = 18) vs. L2 (n = 8) vs. E2 (n = 14) vs. E3 (n = 5)**	**103.5 (72.63–143.00) vs. 63.42 vs. 50.20 (33.99–55.89) vs. 51.70 (41.15–58.28)**	**Kruskal–Wallis test**	**0.015**

CD—Crohn Disease; UC—ulcerative colitis; L1—ileal Crohn Disease, L2—colonic Crohn Disease, L3—ileocolonic Crohn Disease, E1—proctitis, E2—left sided ulcerative colitis, E3—extensive ulcerative colitis.

## Data Availability

The data that support the findings of this study are available from the corresponding author upon reasonable request.
